# Impact of pre-treatment C-reactive protein level and skeletal muscle mass on outcomes after stereotactic body radiotherapy for T1N0M0 non-small cell lung cancer: a supplementary analysis of the Japan Clinical Oncology Group study JCOG0403

**DOI:** 10.1093/jrr/rrab065

**Published:** 2021-08-05

**Authors:** Yukinori Matsuo, Yasushi Nagata, Masashi Wakabayashi, Satoshi Ishikura, Hiroshi Onishi, Masaki Kokubo, Katsuyuki Karasawa, Yoshiyuki Shioyama, Rikiya Onimaru, Masahiro Hiraoka

**Affiliations:** 1 Department of Radiation Oncology and Image-Applied Therapy, Kyoto University, Kyoto, 6068507, Japan; 2 Department of Radiation Oncology, Hiroshima University, Hiroshima, 7348551, Japan; 3 Japan Clinical Oncology Group Data Center/Operations Office, National Cancer Center Hospital, Tokyo, 1040045, Japan; 4 Department of Radiology, Nagoya City University Graduate School of Medical Sciences, Nagoya, 4678601, Japan; 5 Department of Radiology, University of Yamanashi, Yamanashi, 4093898, Japan; 6 Department of Radiation Oncology, Kobe City Medical Center General Hospital, Kobe, 6500047, Japan; 7 Department of Radiology, Tokyo Metropolitan Komagome Hospital, Tokyo, 1138677, Japan; 8 Department of Clinical Radiology, Graduate School of Medical Sciences, Kyushu University, Fukuoka, 8128582, Japan; 9 Department of Radiation Oncology, Tonan Hospital, Sapporo, 0600004, Japan; 10 Department of Radiation Oncology, Japanese Red Cross Society Wakayama Medical Center, Wakayama, 6408558, Japan

**Keywords:** C-reactive protein (CRP), sarcopenia, stereotactic body radiotherapy (SBRT), lung cancer

## Abstract

This study aimed to evaluate the impact of pretreatment C-reactive protein (CRP) and skeletal muscle mass (SMM) on outcomes after stereotactic body radiotherapy (SBRT) for T1N0M0 non-small cell lung cancer (NSCLC) as a supplementary analysis of JCOG0403. Patients were divided into high and low CRP groups with a threshold value of 0.3 mg/dL. The paraspinous musculature area at the level of the 12th thoracic vertebra was measured on simulation computed tomography (CT). When the area was lower than the sex-specific median, the patient was classified into the low SMM group. Toxicities, overall survival (OS) and cumulative incidence of cause-specific death were compared between the groups. Sixty operable and 92 inoperable patients were included. In the operable cohort, OS significantly differed between the CRP groups (log-rank test p = 0.009; 58.8% and 83.6% at three years for high and low CRP, respectively). This difference in OS was mainly attributed to the difference in lung cancer deaths (Gray’s test p = 0.070; 29.4% and 7.1% at three years, respectively). No impact of SMM on OS was observed. The incidence of Grade 3–4 toxicities tended to be higher in the low SMM group (16.7% vs 0%, Fisher’s exact test p = 0.052). In the inoperable cohort, no significant impact on OS was observed for either CRP or SMM. The toxicity incidence was also not different between the CRP and SMM groups. The present study suggests that pretreatment CRP level may provide prognostic information in operable patients receiving SBRT for early-stage NSCLC.

## INTRODUCTION

Sarcopenia and systemic inflammation have received increasing attention as prognostic factors in many types of cancer, either separately [1–3] or in combination [4]. Sarcopenia is defined as ‘a decrease in skeletal muscle mass (SMM) and muscle strength or physical function, such as gait speed, observed in elderly individuals,’ according to the Japanese clinical guidelines for sarcopenia [5]. A meta-analysis by Shachar *et al.* demonstrated that low skeletal muscle index at diagnosis is associated with poor prognosis in patients with solid tumors [2]. Sarcopenia has also been reported to be related to postoperative complication in cancer patients [6]. Aging is a major cause of sarcopenia, but it is not the sole cause. Insufficient physical activity, concomitant diseases, including malignancy and malnutrition, also cause sarcopenia [7].

For systemic inflammation, serum levels of C-reactive protein (CRP) and neutrophil-to-lymphocyte ratio (NLR) derived from complete blood count have been well investigated as prognostic inflammatory markers in cancer patients [8, 9]. Increased levels of inflammatory markers are associated with age and age-related diseases, including sarcopenia and cancer [10]. Thus, aging, cancer, sarcopenia and systemic inflammation are deeply interrelated.

Stereotactic body radiotherapy (SBRT) is a technique for delivering radiation that is characterized by high conformality to the target and a small number of fractions with a large fractional dose. Early-stage non-small cell lung cancer (NSCLC) is commonly treated with SBRT. SBRT achieves a high proportion of primary tumor control of approximately 90% for early-stage NSCLC, and shows improved survival compared to conventional radiotherapy [11]. Several prospective phase II trials have proven the efficacy and safety of SBRT for early-stage NSCLC [12–14]. JCOG0403 is a multicenter prospective phase II trial of SBRT for early-stage NSCLC [14]. Overall survival (OS) at three years was reported to be 76.5% and 59.9% in operable and inoperable patients eligible for analysis, respectively. Toxicities after SBRT were acceptable, though inoperable patients were more prone to severe (Grade 3–4) toxicities than operable patients (12.5% vs 6.2%). Based on these results, SBRT is now recognized as the standard therapy for medically inoperable patients with early-stage NSCLC. Introduction of SBRT contributed to improved survival in elderly patients (≥ 65 years) with clinical stage I NSCLC in the Netherlands [15]. As SBRT is used for patients who are medically inoperable or are at high risk for surgery, the age of patients receiving SBRT tends to be higher [16]. Such elderly patients are likely to have systemic inflammation and sarcopenia. Association of systemic inflammation or sarcopenia with outcomes after SBRT for early-stage NSCLC was previously investigated at Kyoto University. Kishi *et al.* retrospectively evaluated the modified Glasgow Prognostic Score (mGPS) as a prognosticator in 165 patients with stage I NSCLC treated with SBRT [17]. The mGPS is an inflammation-based prognostic score consisting of serum CRP and albumin levels. In the Kishi‘s study, CRP mainly contributed to mGPS. OS was significantly different between the mGPS groups (66.4% vs 44.5% at three years for the low and high mGPS, p < 0.001). The high incidence of lung cancer death in patients with high mGPS led to worse OS. For sarcopenia, Matsuo *et al.* measured SMM of the psoas muscle at the level of the third lumbar vertebra (L3) in 186 patients treated with SBRT for stage I NSCLC [18]. Low SMM was associated with a high incidence of non-lung cancer death, but not with the incidence of lung cancer death. Based on these experiences, we hypothesized that sarcopenia and systemic inflammation might have a greater impact on prognosis in elderly patients undergoing SBRT, especially systemic inflammation associated with lung cancer death, and sarcopenia with non-lung cancer death.

In JCOG0403, pretreatment blood test data, which included CRP but not albumin, and computed tomography (CT) images for treatment planning were prospectively collected, which enabled us to evaluate inflammation and SMM. The purpose of this study was to evaluate the impact of pretreatment CRP and SMM on outcomes after SBRT for T1N0M0 NSCLC using blood and CT data as a supplementary analysis of JCOG0403.

## MATERIALS AND METHODS

Details of the JCOG0403 were available in the report by Nagata *et al.* [14]. Medically inoperable and operable patients with clinical T1N0M0 (Union for International Cancer Control staging criteria, the 6th edition, 2002) NSCLC were separately enrolled to JCOG0403. The primary endpoint was three-year OS for both inoperable and operable patients. A total of 169 patients (104 inoperable and 65 operable patients) were registered. The patients underwent SBRT with a prescribed dose of 48 Gy in 4 fractions at the isocenter. A blood test, including CRP, was mandatory within 14 days before registration of a patient to the trial. There was no eligibility criterion for the serum CRP value for JCOG0403. Pretreatment CRP of all registered patients was recorded in a case report form. Before SBRT delivery, three-dimensional treatment planning was performed using thoracic CT images acquired for the SBRT simulation. The slice thickness of the simulation CT was 1–3 mm around the tumor level, and 10 mm or less elsewhere. The simulation CT images were collected after the completion of SBRT for quality assurance of the treatment. The treatment as per protocol had to be initiated within 10 days of registration. The overall treatment time was around four to eight days, but was allowed up to a maximum of 15 days.

The present study used the same data set as in the previous report with the data cutoff of December 2011 [14]. The present study excluded patients who were ineligible for JCOG0403 protocol and patients in whom the paraspinal muscle area could not be measured on the simulation CT images. Patients were divided into high and low CRP groups with a threshold value of 0.3 mg/dL. A cross-sectional area of the paraspinal muscles at the mid-level of the 12th thoracic vertebra was contoured on the simulation CT by an experienced radiation oncologist (Y.M.), and extracted with thresholding Hounsfield Units between −29 and 150 [19]. Normalization of the cross-sectional area according to the patient height could not be done because the height data were not collected for JCOG0403. When the cross-sectional area was lower than the sex-specific median, the patient was classified as having a low SMM.

The present study evaluated the operable and inoperable cohorts separately because they were different in proportions of survival and toxicity. OS, cumulative incidence of cause-specific death, and proportion of Grade 3 or worse toxicities were compared between the groups. The Kaplan–Meier method and cumulative incidence function were used to estimate the proportion of OS and cumulative incidence of cause-specific death, respectively. A log-rank test and Gray’s test were used to compare OS and cumulative incidence, respectively. Fisher’s exact test was used to compare patient characteristics or the proportion of toxicities between two groups. A two-sided *p*-value <0.05 was considered statistically significant.

## RESULTS

**Fig. 1:**
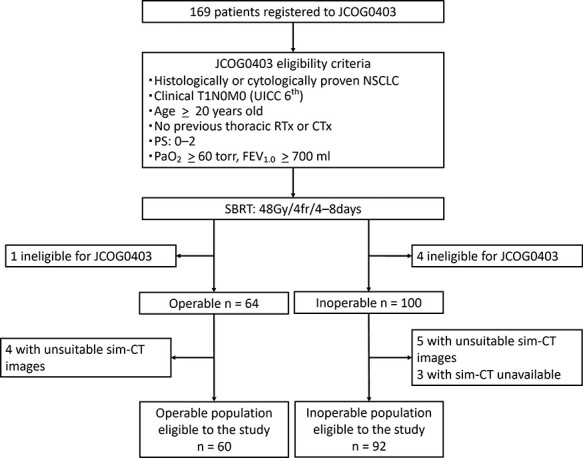
Study design. Abbreviations: JCOG, Japan Clinical Oncology Group; NSCLC, non-small cell lung cancer; UICC, Union for International Cancer Control; RTx, radiotherapy; CTx, chemotherapy; PS, performance status; SBRT, Stereotactic body radiotherapy; PaO_2_ arterial partial pressure of oxygen; FEV_1.0_, forced expiratory volume in one second; sim-CT, computed tomography for simulation.

Sixty operable and 92 inoperable patients were included in the present study after excluding five patients ineligible for JCOG0403, three patients whose simulation CTs were unavailable, and nine patients whose simulation CTs were unsuitable for the SMM measurement due to noise on the CT images (Table 1 and Fig. 1). The median follow-up period was 67 months (range, 58–72 months) and 47 months (range, 39–57 months) for the operable and inoperable cohorts, respectively.

**Table 1:** Patient characteristics

	Operable (*n* = 60)	Inoperable (*n* = 92)
**Age [y], median (range)**	79 (54–91)	78 (59–90)
**Sex**		
Male	41	67
Female	19	25
**PS**		
0	40	43
1	18	41
2	2	8
**Tumor size [mm], median (range)**	21.5 (10–30)	21 (9–30)
**Histology**		
Squamous	18	36
Adeno	38	44
Others	4	12
**CRP [mg/dL], median (range)**	0.1 (0.0–7.3)	0.2 (0.0–5.5)
SMM^*^ [cm ^**2**^ **], median (range)**		
Male	31.5 (16.3–50.4)	31.8 (12.6–52.9)
Female	25.6 (13.9–32.6)	24.8 (3.4–38.5)

Abbreviations: CRP, C-reactive protein; SMM, skeletal muscle mass; PS, performance status; Squamous, squamous cell carcinoma; Adeno, adenocarcinoma.

^^*^^SMM is expressed as a cross-sectional area of the paraspinal muscles at the mid-level of the 12th thoracic vertebra

Median CRP level was 0.2 mg/dL (range, 0.0–7.3 mg/dL). Forty-two patients (28.3% and 27.2% in the operative and inoperative cohorts, respectively) were classified as having high CRP. The medians of the cross-sectional area of the paraspinal muscles in the entire patient cohort were 31.6 cm^2^ (range, 12.6–52.9 cm^2^) and 25.1 cm^2^ (range, 3.4–38.5 cm^2^) in males and females, respectively. The proportion of patients with low SMM was 50% in both the operable and inoperable cohorts.

### Operable cohort

In the operable cohort (Table 2), patients with high CRP tended to be elderly. A performance status of 0 was significantly less frequent in the low SMM group. CRP and SMM status tended to correlate with each other.

**Table 2:** Comparison of patient characteristics of the operable cohort

Operable cohort	CRP			SMM		
	Low (*n* = 43)	High (*n* = 17)	*p value*	High (*n* = 30)	Low (*n* = 30)	*p value*
**Age [y], median (range)**	78 (54–91)	80 (73–87)		78 (54–91)	80 (61–87)	
≤ 75 y	17	2	*0.095*	13	6	*0.177*
76–80 y	14	7		9	12	
≥ 81 y	12	8		8	12	
**Sex**						
Male	28	13	*0.541*	19	22	*0.580*
Female	15	4		11	8	
**PS**						
0	30	10	*0.480*	24	16	*0.006*
1	12	6		4	14	
2	1	1		2	0	
**Smoking**						
No	13	5	*1.000*	12	6	*0.158*
Yes	30	12		18	24	
**BW loss for 6 m**						
≤ 5%	33	14	*0.323*	23	24	*0.404*
> 5%	2	2		1	3	
NA	8	1		6	3	
**Tumor size [mm], median (range)**	21 (10–30)	24 (15–30)		23 (12–30)	20.5 (10–29)	
≤ 20 mm	21	7	*0.775*	13	15	*0.796*
21–30 mm	22	10		17	15	
**Histology**						
Squamous	12	6	*0.426*	9	9	*1.000*
Adeno	29	9		19	19	
Others	2	2		2	2	
**CRP**						
< 0.3 mg/dL	43	–		25	18	*0.084*
≥ 0.3 mg/dL	–	17		5	12	
**SMM**						
High	25	5	*0.084*	30	–	
Low	18	12		–	30	

Abbreviations: CRP, C-reactive protein; SMM, skeletal muscle mass; PS, performance status; BW, body weight; Squamous, squamous cell carcinoma; Adeno, adenocarcinoma.

**Fig. 2:**
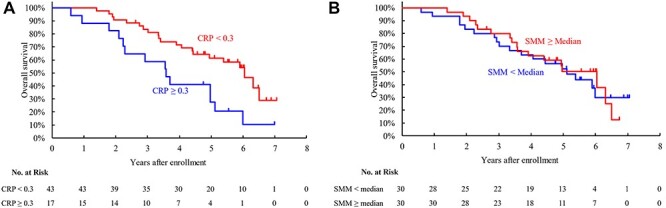
Overall survival in the operable cohort according to the CRP (a) and SMM (b) groups. Abbreviations: CRP, C-reactive protein; SMM, skeletal muscle mass.

The three-year OS was 58.8% (95% CI, 32.5–77.8%) and 83.6% (95% CI, 68.6–91.8%) for high and low CRP, respectively. The difference in OS between the CRP groups was significant (log-rank test p = 0.009; hazard ratio [HR] of high CRP, 2.43 [95% confidence interval (CI), 1.23–4.80], Fig. 2a). The cumulative incidence of lung cancer death tended to be higher in the high CRP group than in the low CRP group (Gray’s test p = 0.070, Supplementary Fig. 1). The cumulative incidence of lung cancer death at three years was 29.4% (95% CI, 10.1–52.0%) and 7.1% (95%CI, 1.8–17.5%), respectively. The cumulative incidence of non-lung cancer death did not differ between the CRP groups (11.8% vs 9.3% at three years, Gray’s test p = 0.623). No impact of the SMM status on OS was observed in the operable patients (73.3% vs 79.9% at three years, log-rank test p = 0.834, Fig. 2b).

**Fig. 3:**
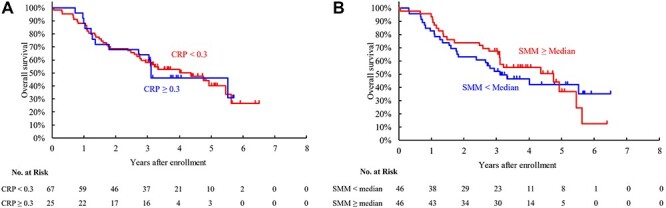
Overall survival in the inoperable cohort according to the CRP (a) and SMM (b) groups. Abbreviations: CRP, C-reactive protein; SMM, skeletal muscle mass.

Grade 3–4 toxicities were observed in 17.7% and 4.7% of patients with high and low CRP, respectively (p = 0.132). The incidence of Grade 3–4 toxicities tended to be higher in the low SMM group (16.7% vs 0%, p = 0.052).

### Inoperable cohort

In the inoperable cohort (Table 3), the high CRP level was associated with male sex, body weight loss and larger tumor size. No association was observed between CRP and SMM status.

**Table 3:** Comparison of patient characteristics of the inoperable cohort

Inoperable cohort	CRP			SMM		
	Low (*n* = 67)	High (*n* = 25)	*p value*	High (*n* = 46)	Low (*n* = 46)	*p value*
**Age [y], median (range)**	78 (59–87)	77 (67–90)		76 (60–89)	79 (59–90)	
≤ 75 y	22	10	*0.838*	20	12	*0.168*
76–80 y	23	8		15	16	
≥ 81 y	22	7		11	18	
**Sex**						
Male	44	23	*0.016*	35	32	*0.640*
Female	23	2		11	14	
**PS**						
0	33	10	*0.609*	23	20	*0.444*
1	29	12		21	20	
2	5	3		2	6	
**Smoking**						
No	15	2	*0.141*	6	11	*0.283*
Yes	52	23		40	35	
**BW loss for 6 m**						
≤ 5%	57	16	*0.019*	38	35	*0.648*
> 5%	6	2		4	4	
NA	4	7		4	7	
**Tumor size [mm], median (range)**	20 (9–30)	24 (11–30)		20 (9–30)	22 (11–30)	
≤ 20 mm	39	7	*0.018*	27	19	*0.144*
21–30 mm	28	18		19	27	
**Histology**						
Squamous	25	11	*0.723*	16	20	*0.681*
Adeno	32	12		24	20	
Others	10	2		6	6	
**CRP**						
< 0.3 mg/dL	67	–		32	35	*0.640*
≥ 0.3 mg/dL	–	25		14	11	
**SMM**						
High	32	14	*0.640*	46	–	
Low	35	11		–	46	

Abbreviations: CRP, C-reactive protein; SMM, skeletal muscle mass; PS, performance status; BW, body weight; Squamous, squamous cell carcinoma; Adeno, adenocarcinoma.

OS did not differ between the CRP groups (64.0% vs 58.1% at three years for the high and low CRP, log-rank test p = 0.925, Fig. 3a). No significant difference was observed in OS between the SMM groups (52.0% vs 67.4% at three years for the low and high SMM, p = 0.751, Fig. 3b). There was no significant impact of CRP or SMM on lung cancer death or non-lung cancer death (Supplementary Fig. 2). Toxicity incidence was not different between the CRP and SMM groups (Grade 3–4 incidence, 20% and 14.9% for the high and low CRP groups, p = 0.541; 17.4% and 15.2% for the low and high SMM groups, p = 1.000, respectively).

## DISCUSSION

The present study suggests that CRP and SMM may provide information on survival and toxicities after SBRT for early-stage NSCLC in operable patients. To the best of our knowledge, this is the first study to evaluate sarcopenia and systemic inflammation in SBRT for early-stage NSCLC using prospective data. The present study was conducted as a supplementary analysis of JCOG0403 based on the experiences of one of the participating institutions [17, 18]. The strength of this study is the use of a prospective cohort that is rigorously managed by an experienced data center. Most similar studies on sarcopenia and/or inflammation in cancer patients have been conducted in a retrospective manner.

CRP is a marker of acute-phase inflammatory response. Although the exact mechanisms for CRP to be associated with worse prognosis in cancer patients are still unclear, CRP might reflect malignant characteristics of the tumor. Baseline CRP level have been studied as a prognostic factor in early-stage NSCLC in multiple studies. A meta-analysis conducted by Leuzzi *et al.* based on eight surgery papers and two SBRT papers found that elevated pretreatment CRP levels were significantly associated with poor OS (HR, 1.60; 95% CI, 1.30–1.97; p < 0.001) [20].

CRP is not specific to cancer, but it is also related to non-cancer diseases. Minor elevation of CRP is related to genetic, demographic and dietary characteristics as well as non-cancer diseases, either cardiovascular or non-cardiovascular [21]. The negative impact of CRP on survival is not limited to cancer but is observed in cardiovascular disease, diabetes mellitus, chronic obstructive pulmonary disease, etc. [10]. Therefore, CRP should be associated with non-lung cancer death as well as lung cancer death. However, in the inoperable cohort, CRP did not show any significant association with either of the two types of deaths. We could not clearly explain the reasons for the negative CRP results in the inoperable patients. Non-lung cancer mortality was considerably high (>30% at five years) in the inoperable patients, which might have diminished the negative effect of CRP on survival.

There are four reports available on the effect of inflammatory markers other than CRP on survival in patients treated with SBRT for early-stage NSCLC [22–25]. The four reports used two or three pretreatment inflammation markers based on complete blood count: NLR, platelet-to-lymphocyte ratio and monocyte-to-lymphocyte ratio. Although threshold values varied slightly among the reports, the association of higher NLR with worse OS was a common finding in all the four studies. NLR reflects neutrophilia and/or lymphopenia, and is known to be a prognostic factor in cancer patients [9]. There is no rigorous evidence on which CRP or NLR performs better in prognostication in cancer. A few reports that evaluated both CRP and NLR suggested that these might be independent prognostic factors [26, 27]. More research is needed to elucidate which of the multiple inflammatory markers should be used or in combination.

The advantage of SMM in sarcopenia research is that it can be easily quantified from CT scans, which are routinely acquired for staging of cancer [2]. However, there are two major issues in the sarcopenia research in the cancer field. First, no consensus is available on the measurement method of SMM or on the definition of low SMM. The total psoas cross-sectional area at the L3 level and the total abdominal muscle area at the L3 level are the most common indices for SMM measurement [28]. A few studies used the cross-sectional area of the paraspinal muscle at the Th12 level, similar to the present study, which was reported to be associated with the psoas muscle area [29]. The area values were normalized by dividing by the square of the height in most studies. Absence of normalization might have affected the results in the present study. Second, most studies on sarcopenia in the cancer field evaluate only muscle mass, and they lack muscle function evaluation. As described above, loss of SMM is a key component of sarcopenia. However, it is insufficient for the diagnosis of sarcopenia. Although the definition of sarcopenia varies slightly among guidelines, deterioration of muscle function is another important prerequisite in most sarcopenia guidelines [5]. Both muscle mass and muscle function decline with increasing age, but the latter declines faster, especially in octogenarians [30]. Poor muscle function associated with mortality more strongly than low muscle mass does [31]. The present study included patients who were older than 80 years of age. This might be the reason why SMM alone did not work well as a prognosticator in the present study. Future studies regarding sarcopenia in patients with cancer should include both muscle mass and muscle function.

Systemic inflammation and sarcopenia are important with regard to safety and efficacy, not only in SBRT patients, but also in those who use immune checkpoint inhibitor (ICI) in combination with SBRT. ICIs have recently received much attention because of their potential synergistic effect when combined with SBRT [32]. SBRT provides a high proportion of local tumor control of as high as 90%, but regional recurrence and distant metastasis are major issues after SBRT. In JCOG0403, approximately 30% of the patients developed regional lymph node recurrence and/or distant metastasis. Systemic therapy that is tolerable in elderly patients who receive SBRT is needed. ICI combined with SBRT for early-stage NSCLC is now under investigation as an adjuvant systemic therapy for SBRT patients. Recently, Chang *et al.* reported the interim analysis of adverse events in the I-SABR trial that evaluated nivolumab, a kind of ICI, combined with SBRT for early stage or isolated local recurrence of NSCLC [33]. Ninety-two patients with a median age of 72 years (range, 57–90 years) were randomized into SBRT alone or combined ICI. They concluded that nivolumab immunotherapy combined with SBRT appeared to be well-tolerated in fragile patients. Systemic inflammation and sarcopenia are now examined in patients with advanced NSCLC as prognosticators or predictors in ICI treatment by several researchers [34–36]. Systemic inflammation and sarcopenia may be worth considering in patients treated with SBRT plus ICI.

The limitation of the study is the lack of data that other similar studies evaluated, including patients’ height, muscle function, albumin and NLR. These data were not collected because they were not supposed to be related to eligibility criteria or primary endpoint at the time of planning of JCOG0403. The other limitation is that the present study is based on a single trial using 48 Gy in 4 fractions. Further evaluation is needed to know if the results from this study can be applied to patients treated with other fractionation schemes, such as 45 Gy in 3 fractions [12] or 54 Gy in 3 fractions [13].

In conclusion, the present study suggests that CRP may provide prognostic information for operable patients receiving SBRT for early-stage NSCLC. Further studies are warranted to confirm this finding.

## CONFLICT OF INTEREST

The authors declare they have no conflict of interest.

## FUNDING

This work was supported in part by the National Cancer Center Research and Development Fund (23-A-16, 23-A-21, 26-A-4, 29-A-3 and 2020-J-3); a Grant-in-Aid for Cancer Research (14S-4, 17S-5, 20S-5 and 20S-6); a Health and Labor Sciences Research Grant for Clinical Cancer Research (H15–41, H18–014) from the Japanese Ministry of Health, Labor and Welfare; and AMED under Grant Number JP20ck0106581.

## PRESENTATION AT A CONFERENCE

This work was presented at the 17th World Conference on Lung Cancer held during December 4–7, 2016.

## CLINICAL TRIAL REGISTRATION NUMBER

UMIN-CTR, C000000029.

## Supplementary Material

Supplementary_Data_rrab065
